# Cellular Stress Response and Immune Signaling in Retinal Ischemia–Reperfusion Injury

**DOI:** 10.3389/fimmu.2016.00444

**Published:** 2016-10-24

**Authors:** Gillipsie Minhas, Jyoti Sharma, Nooruddin Khan

**Affiliations:** ^1^Department of Biotechnology and Bioinformatics, School of Life Sciences, University of Hyderabad, Hyderabad, Telangana, India

**Keywords:** retina, ischemia–reperfusion, inflammation, immune response, stress response

## Abstract

Ischemia–reperfusion injury is a well-known pathological hallmark associated with diabetic retinopathy, glaucoma, and other related retinopathies that ultimately can lead to visual impairment and vision loss. Retinal ischemia pathogenesis involves a cascade of detrimental events that include energy failure, excitotoxic damage, calcium imbalance, oxidative stress, and eventually cell death. Retina for a long time has been known to be an immune privileged site; however, recent investigations reveal that retina, as well as the central nervous system, elicits immunological responses during various stress cues. Stress condition, such as reperfusion of blood supply post-ischemia results in the sequestration of different immune cells, inflammatory mediators including cytokines, chemokines, etc., to the ischemic region, which in turn facilitates induction of inflammatory conditions in these tissues. The immunological activation during injury or stress *per se* is beneficial for repair and maintenance of cellular homeostasis, but whether the associated inflammation is good or bad, during ischemia–reperfusion injury, hitherto remains to be explored. Keeping all these notions in mind, the current review tries to address the immune response and host stress response mechanisms involved in ischemia–reperfusion injury with the focus on the retina.

## Introduction

Retinal ischemia is a condition that has been found to be connected to a large number of retinal diseases such as glaucoma, diabetic retinopathy, and central retinal artery occlusion, which are a leading cause of visual impairment or blindness (1–3). Ischemia, in general, is a condition that occurs due to disruption in blood supply to a particular tissue or organ, which cuts the supply of oxygen and glucose, triggering a cascade of events that ultimately ends with cell death. Retina being highly metabolic has very high oxygen consumption in the body (4). It is sensitive to oxygen deficiency, thus making it more susceptible to ischemic injury. Retina being an extension of central nervous system (CNS), it makes retina an ideal model system not only to examine the pathophysiology behind ischemia/hypoxia but also to assess different therapeutic strategies in animal models before proceeding to clinical trials, which can also be extrapolated to the brain (5, 6).

Ischemic cascade consists of energy failure, calcium influx followed by depolarization, and oxidative stress (7). Inflammation is an important phenomenon in the progression of any injury including ischemic injury (8). It usually helps in repair mechanism, but chronic inflammation causes more damage than good, triggering the release of reactive oxygen species (ROS), and tissue destruction (9). In ischemia–reperfusion injury, a similar condition is observed during the reperfusion, which is the restoration of blood supply to the ischemic tissue. The reoxygenation of tissue after ischemia causes more destruction by the production of ROS that damage the biomolecules by activation of inflammatory responses (10). In the current review, we have focused on the immune processes observed during ischemia–reperfusion injury, especially in the retina.

## Retina: Immune Privileged Site

For a long time, retina and CNS have been considered as an immune privileged site due to its inability to post an immune response. The immune cells, which play the usual function of processing and presenting the antigens in the periphery, to our knowledge have not been reported in the retinal or CNS tissues; as a result, CNS is unable to mount an adaptive immune response (11). Investigations have also shown the presence of elevated levels of anti-inflammatory cytokines, which makes the natural environment in the brain as anti-inflammatory (12). Eye also has its similar mechanisms to control immune activation, and it contains many factors in aqueous humor that have shown to decrease the IFNγ production through the presence of anti-inflammatory factors (e.g., TGFβ2), which has demonstrated to reduce TCR activation (13–15).

The blood–brain barrier (BBB) provides anatomical and physiological protection to CNS. It restricts the migration of T lymphocytes and other immune mediators to brain through tight junctions between endothelial cells and helps in the maintenance of CNS as a unique immune privileged site (16, 17). On the similar lines, blood–retina barrier (BRB) is known to maintain homeostasis in the retina, which is essential for maintenance of immune privilege in the eye (18). The structure of BRB allows sustaining this condition in the eye. It is composed of two layers of tight junctions: the inner junction is present between retinal capillary endothelial cells and the outer one is between the RPE (18, 19). Many factors have been studied which affect the permeability of BRB, such as oxidative stress, VEGF, and inflammation (20, 21).

**Figure 1:**
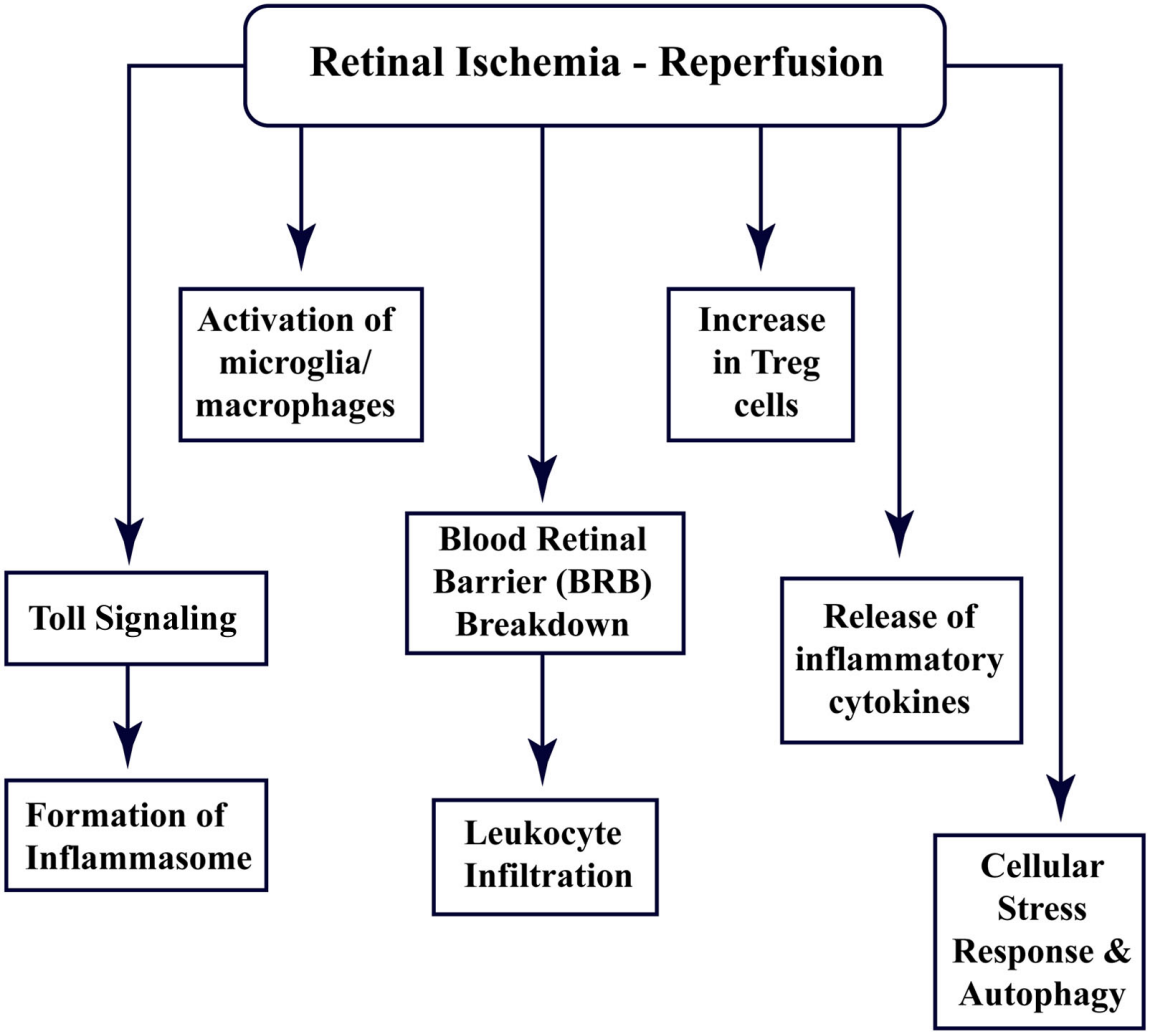
**Multi-faceted cellular responses during retinal ischemia–reperfusion injury**. Different aspects of immune signaling and host responses associated with ischemia–reperfusion injury in retina.

Recent studies have shown that the CNS tissues have specific immune responses against different kinds of trauma, infection, or injury, and the term “immune privilege” has become implicit (22). All the abovementioned factors and barriers make the CNS and retinal tissue isolated from the immune response; however, still, these components interact with the peripheral immune system (15, 23). Figure 1 enlists different aspects of cellular and immune response associated with retinal ischemia–reperfusion injury, which have been explored through this review.

## Ischemia–Reperfusion: Sterile Inflammation

Inflammation is a crucial component of host immune response essential for defense against invading pathogens, and it involves activation of different immune cells and the release of cytokines, chemokines, and other effector molecules. However, there are injuries which do not include any pathogen invasion but still invoke an inflammatory response, such as ischemia and trauma; these are identified as *sterile inflammation* (24).

In the case of the microbe-induced inflammation, it is activated through pattern-recognition receptors (PRRs), and these receptors recognize different pathogen-associated molecular patterns (PAMPs), such as lipopolysaccharide (LPS), dsRNA, toxins, and other foreign molecules. In contrast, sterile inflammation involves damage-associated molecular patterns (DAMPs) (24). The DAMPs operate in the same way as the PAMPs but are endogenous instead of being pathogen derived. DAMPs are released by necrotic or apoptotic cells during injury and include proteins such as high-mobility group box-1 (HMGB1), mitochondrial components, uric acid, and others (25). Both PAMPs and DAMPs act through toll-like receptors (TLRs), NOD-like receptors (NLRs), and C-type lectin receptors, which are the common PRRs that are present on immune cells (26).

## Toll-Like Receptor Signaling

**Figure 2:**
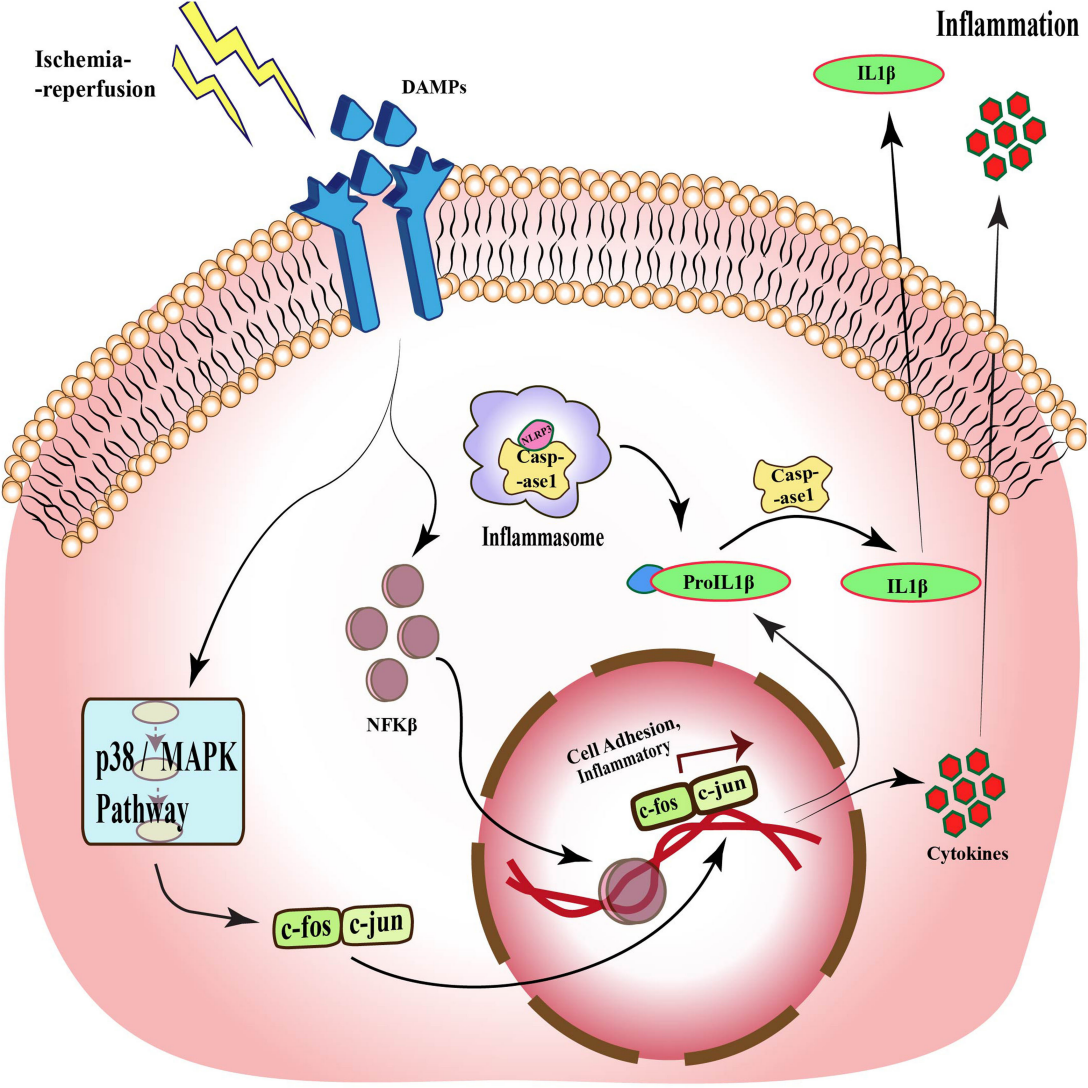
**Toll-like receptor signaling during ischemia–reperfusion injury**. Specific receptors, such as TLRs, are activated during ischemia–reperfusion through damage-associated molecular patterns (DAMPs), which activate downstream MAPK pathway and *via* different transcription factors such as NFκB and c-fos-c-jun, and translate different cell adhesion and inflammatory molecules, which causes inflammation. This pathway also acts through inflammasomes formation and through caspase 1 activation, which also results in inflammation.

Toll signaling is a pathway that is known to be activated in response to many diseases such as ischemia. This pathway was first identified in *Drosophila*, where it is essential for embryonic development (27–29). Subsequent studies demonstrated its presence in mammals (30, 31). TLRs are evolutionarily conserved membrane proteins with leucine-rich repeats and extracellular ligand-binding domains that recognize the PAMPs and DAMPs (32). TLR4 was the first component to be identified in mammals, which showed activation by bacterial components. These bacterial cell wall components upon binding to TLR activate the release of cytokines that assist in clearance of microbes, but in the case of excessive activation, it can become detrimental to host cells (33). Although it is now well established that TLR activation is associated with pathogen invasion, TLR has also been shown to be activated by DAMPs released by cells under stress. One such molecule is HMGB1, which is released by necrotic or apoptotic cells (34). Activation of TLR signaling, in turn, stimulates NFκB, a transcription factor that regulates the expression of genes responsible for cell adhesion, innate immune response, and inflammation (35, 36). Figure 2 shows the activation of TLR upon stimulation by DAMPs and downstream signaling pathways in response to ischemia–reperfusion, which through p38/MAPK signaling and NFκB leads to expression of inflammatory cytokines and activation of inflammasomes (37).

Studies from different animal models have shown the important role of TLR signaling in ischemia–reperfusion injury (38–42). Hua et al. investigated TLR signaling in global cerebral ischemia and revealed neuronal death and increased expression of proinflammatory cytokines in wild-type mice subjected to ischemic injury. In the absence of TLR4, less infarct volumes with reduced cell death were observed in the cerebral ischemia mouse model, which demonstrates the deleterious role for TLR in ischemia–reperfusion injury (41). Lehnardt et al. showed similar outcome with TLR2-deficient mice, which developed less injury as compared to wild-type subjected to middle cerebral artery occlusion (MCAO) (43). More studies done to delineate the mechanism behind the protective role of TLR deletion have shown the contribution of PI3K/Akt pathway, which is already known to prevent apoptosis (44, 45). The outcomes observed in the brain were also reflected in retinal ischemia–reperfusion injury. Qi et al. generated retinal ischemia injury in rats by clamping retinal arteries and demonstrated the TLR4 activation post-injury (46). Studies have shown that TLR4-deficient mice are neuroprotective in ischemia–reperfusion (47). Kilic et al. exhibited less ischemic damage post-ischemia in focal cerebral ischemia as well as retinal ganglion cells (RGCs) degeneration in a TLR4 knock-out model (48). He et al. also demonstrated attenuation in neovascularization in retina along with less microglial activation and proinflammatory cytokines levels in TLR4-deficient mice subjected to ischemia (49). These studies emphasize the TLRs as prospective therapeutic targets to regulate ischemia–reperfusion injury.

## Activation of Inflammasomes

Inflammasomes are the component of innate immune response, which have been implicated in different metabolic and neurodegenerative diseases. Inflammasomes are intracellular, multimeric protein complexes that are formed post-detection of PAMPs or DAMPs by specific PRRs. Inflammasome activation stimulates the expression of IL1β, IL18, and other proinflammatory cytokines downstream to initiate the cascade through caspase 1 (50, 51). Mechanistically, inflammasomes recruit pro-caspase 1, which further oligomerizes and auto-cleaves to form active caspase 1 that cleaves the pro-forms of cytokines IL1β and IL18 into their active forms (52).

The inflammasomes are generally named based on the scaffold protein that is associated with it. The most common being the NLRs, which also belong to the family of PRRs and are analogous to TLRs (53). The best-investigated inflammasomes that have been found to be associated with neuroinflammation are NACHT domain-LRR domain and pyrin domain containing protein (NALP) and nucleotide-binding oligomerization domain-like receptor with pyrin domain protein (NLRP1 and 3) (54, 55). The activation of NLRP inflammasomes through TLR4 activation has been demonstrated in a retinal ischemia–reperfusion injury model induced by ligation of retinal blood vessels in rat (46). Chi et al. also investigated the role of inflammasomes in RGC death due to retinal ischemia–reperfusion injury in a caspase 1-independent pathway. The study revealed that the ischemic injury increases the levels of TLR4, which further stimulates IL1β production through caspase 8 pathway (56). These revelations make the inflammasome activation a probable therapeutic for retinal ischemic injuries that will help to regulate inflammatory responses. It has been demonstrated in an *Nlrp3* knock-out mice that the absence of inflammasomes delayed the progression of CNS injury (57).

## Activation of Microglia–Macrophages

Microglia and astrocytes are the resident immune cells present in CNS. These are the first line of defense against any injury or infection in the CNS. The role of microglia and macrophages is controversial with different studies supporting the impact of their activation after the injury (58–60). Studies have shown that the activation of these cells can either be beneficial or detrimental for a tissue. As is seen in the case of Alzheimer’s, where the activation of microglia helps in clearance of amyloid load and hence is beneficial (61, 62). Similarly, activated macrophages assist in removal of leukocytes through phagocytosis (63). In retina, Müller glia are the principal glial cells. Müller glia are the radial glia, which are found throughout the thickness of the retina and are associated with a majority of retinal degenerations. In normal physiology, Müller glia maintain the layer arrangement in the retina, provide trophic support, and remove the waste, whereas under the pathological conditions, these cells undergo reactive gliosis. The involvement of Müller glia in ischemic injury has been implicated through different animal models, and it has been shown to play protective as well as destructive role in retinal degenerations (64–67). Wurm et al. investigated the impact of ischemia–reperfusion injury on the Müller glial cells in porcine retina exposed to high intraocular pressure (IOP). The authors demonstrated swelling and gliosis through increased expression of glial fibrillary acidic protein (GFAP) and vimentin in Müller glia (68).

Rangasamy et al. have shown the activation of microglia and macrophages in an *in vivo* as well as the *in vitro* model of diabetic retinopathy, another ischemia-associated condition (69). In *in vitro*, the authors exposed the retinal endothelial cells to high glucose conditions and demonstrated an increase in expression levels of macrophage marker and chemokine ligand (CCl2). A similar response was observed in the rat model of diabetes with elevated macrophage and microglia in the retina (69). The activated glia after ischemic injury releases cytokines, such as VEGF, which assist in angiogenesis and resupply of blood (70). Microglia can also produce neurotrophic factors for cell survival. On the contrary, microglia can also release TNFα and IL1β and aggravate the neuronal damage (71, 72). The role of glial cells in the ischemic injury still needs additional investigations to target them as potential therapeutics.

## Infiltration of Leukocytes: Breach in Blood–Retina Barrier

**Figure 3:**
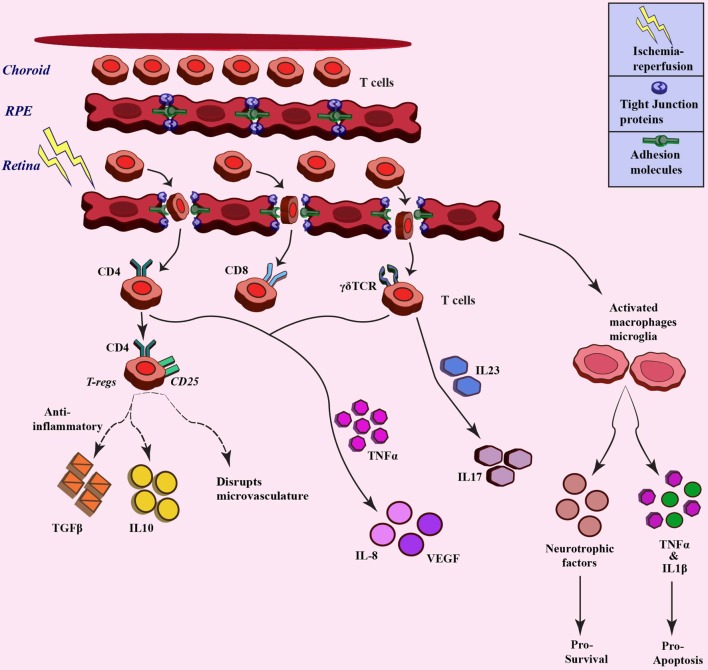
**Trafficking of T cells after ischemia–reperfusion and related downstream response**. Different T cell populations involved in ischemia–reperfusion injury pathogenesis. These T cells infiltrate upon breach of blood-retina barrier due to ischemia injury and different populations stimulate varied downstream signaling through cytokine/chemokine mediators (broken lines denotes the paradox outcomes).

Ischemia–reperfusion injury also has an impact on microcirculation in the tissue, which leads to infiltration of inflammatory cells due to increased permeability (73). The inner retinal barrier is known to be disrupted in many retinal diseases including ischemia (20, 74). Wilson et al. visualized leakage in BRB in a rabbit model of IOP-induced ischemia–reperfusion injury through magnetic-resonance imaging (75). The ischemia–reperfusion injury also impacts the permeability by affecting the different tight-junction proteins that constitute the BRB, such as occludin and zona occludens. Muthusamy et al. in their study in IOP-induced retinal ischemia model in rats have investigated the tight-junction proteins after injury and have shown decreased levels of occludin, which results in the increased vascular permeability and hence infiltration of leukocytes (76, 77). Tsujikawa in their study on rats subjected to optic nerve ligation revealed leukocyte–endothelium interaction through fluorescein angiography (78). Cell adhesion molecules, such as selectins, ICAM1, integrins, and CD11/CD18 have also shown to play a significant role in leukocyte trafficking and infiltration in ischemia (79). Stoll et al. observed an increase in the levels of ICAM and selectin after focal cerebral ischemia, which led to permeation of T cells and macrophages (80). Retinal inflammation has been shown to involve adhesion of leukocytes to the retinal blood vessels, thus affecting the integrity of BRB (69). These molecules can also be exploited as therapy through a decrease in trafficking of immune cells. Tsujikawa et al. in their another study blocked adhesion molecules using specific antibodies, resulting in less leukocyte accumulation during ischemia–reperfusion injury (81). As already discussed in previous sections, reperfusion of blood not only increases oxygen and glucose but also exacerbates the ischemic injury by activating inflammatory responses and promoting immune cells infiltration (9, 82). Lymphocytes, a sub-population of leukocytes, have also been associated with ischemic injury (83). Studies have demonstrated accumulation of T-cells (CD4^+^ and CD8^+^ cells) at the site of ischemic injury in the brain, where different knock-out mice were used to investigate the role of T lymphocytes in cerebral ischemia (84). T lymphocytes have shown their presence at as early as 24 h after ischemic injury, which peaked at day 3 after the injury (85). When cerebral ischemic injury was induced in SCID or Rag^−/−^ mice, which lack the T- and B-cells, less damage and smaller infarct volumes were observed (84). Involvement of T-cells, especially the IL17-producing cells, has also already been demonstrated in different studies in the brain (86, 87). In cerebral ischemia, it has been observed that the γδT cells, a distinct T cell type, are the major IL17-producing cells (88), and the activation of these cells is the primary response to ischemia, which further activates matrix metalloproteinase, pro-inflammatory cytokines, and chemokines that are involved in aggravation of the ischemia–reperfusion injury. A decrease in infarct volume has been demonstrated in TCR-γδ knock-out mice subjected to ischemia (89). Shichita et al. in their study also highlighted a central role of γδT cells in the pathology of cerebral ischemia (90). These studies indicate that targeting T cells could be a novel therapeutic strategy for constraining inflammation during ischemia–reperfusion injury. Apart from these studies, investigations are also required to elucidate the role of T cells-mediated immune modulation during retinal ischemia. Figure 3 gives a brief overview of leukocyte trafficking due to breach in blood–retina barrier after ischemic injury to retina, along with the proposed mechanism about how different subtypes of T cells are involved in ischemia, such as CD4^+^ T regulatory cells that release anti-inflammatory TGFβ, IL10 (91), and γδT cells that produce IL17 through IL23 stimulation (92), along with the production of IL8 and VEGF from different T-cell subtypes (93).

## Protector or Promoter: Regulatory T Cells

Balance of immune responses is essential not only to clear the pathogens but also to control unwanted immune response. Regulatory T cells (Tregs) are the cells which were first identified in the mid-1990s as the cells that control the immune responses through feedback mechanisms (94, 95). These cells also protect the host against any self-antigens and any related auto-immune disease; however, later, it was identified that these cells also have a role to play in the suppression of infections, including uveoretinitis (96). The CD4^+^ T cells principally can be divided into two sub-populations, Th cells that activate immunity and Treg cells that keep a check on the Th cells activity. Treg cells are more precisely identified as the CD4^+^ CD25^+^ population and are characterized by the presence of transcription factor, fork-head box protein 3 (Foxp3) (97, 98). In the case of ischemia, there have been studies with paradox outcomes. Liesz et al. demonstrated a neuroprotective role of Tregs in stroke, with less infarct volumes accompanied by a decrease in levels of proinflammatory cytokines. The authors depleted the Treg cell population and observed an increase in secondary damage to brain post-ischemia along with a higher number of infiltrating leukocytes and activated microglia (99). Treg cells also secrete anti-inflammatory cytokines, such as IL10 and TGFβ. On the contrary, Stubbe et al. in their study on brain subjected to MCAO-induced ischemia reported the detrimental role of Treg cells (100). Kleinschnitz demonstrated that Tregs promote the ischemic injury and impact the microvasculature in the brain. The authors tested the effect of injury in depletion of regulatory T cells (DEREG) mice, which are devoid of any Tregs and revealed less infarct volumes after MCAO (101). Consequently, Treg cells, on the one hand, can protect the brain from ischemic injury by modulating inflammation and increasing the expression of metallomatrix proteases; on the other hand, these cells can also aggravate the ischemic insult by disturbing the microvasculature and hindering functional recovery (102).

Retina-specific Treg cells are produced in response to antigens found in the retina. These can either be natural Tregs, which are generated against retinal antigens expressed in the thymus, or these can be generated independently of thymus from mature peripheral T cells based on exposure to antigens (103). In the retina, this cell population has been associated with uveoretinitis, where an increased number of CD4^+^CD25^+^ Tregs was detected in spleen and eye (104). More studies are required to investigate the role of Tregs in the retina and to translate the findings from cerebral ischemia to the retinal ischemia.

## Molecular Trigger: Inflammatory Cytokines

Cytokines are the molecules released by immune cells after an injury, which can be either anti-inflammatory or proinflammatory in nature. Ischemic injury too is associated with inflammation and release of cytokines. The cytokines induce the migration of leukocytes in the ischemic tissue, which in turn release more cytokines and exacerbate the ischemic injury (105). Both Th1- and Th2-based immune response have been linked with specific proinflammatory cytokine signaling observed in retinal damage. Tumor necrosis factor α (TNF-α) is a proinflammatory cytokine, produced by microglia and leukocytes as an early response toward ischemia–reperfusion. The levels of TNFα were found to be upregulated in porcine retina 5–12 h after ischemia–reperfusion (106). In another model of retinal ischemia produced by an increase in IOP, upregulation in levels of TNF-α as well as its receptors, TNFR1 and TNFR2, was noted as early as 6 h after the reperfusion (107). The increase in TNF-α levels has also been reported by Yoshida et al. in ischemic retina. The expression was found to be localized in macrophages and microglia (108). TNF-α expression further increases the levels of other cytokines, such as IL-8 and VEGF (108, 109). IL17 and IFNγ mRNA levels were also found to be elevated in brain tissue and peripheral blood of permanent MCAO rat models (110). IL17 production has also been identified in human stroke patients (111). IL23 knock-out mice demonstrated smaller infarct volumes (90). IL23 mediates production of IL17 through γδT cells (112). IL6, another Th2 response-related proinflammatory cytokine was detected after reperfusion in high IOP-induced retinal injury in rats (113). Hangai et al. demonstrated many fold increase in IL1α and β levels up to 12 h after ischemia induced by optic nerve ligation in rats (114). These inflammatory cytokines can also be targeted as therapy for ischemia–reperfusion injury. Berger et al. demonstrated improvement in retinal function on treatment with TNFα antibody in high IOP rat model of ischemia–reperfusion (115). Studies have demonstrated protective role of anti-inflammatory interventions in ischemic injury models induced by high IOP as well as related retinal degenerations, such as diabetic retinopathy in rats (116–118).

## Cellular Stress Responses and Immune Regulations During Ischemia

The cellular stress response is the homeostatic mechanism, which enables cells to adapt to various stress cues such as ischemia. Cell senses varieties of stress conditions *via* different sensors such as general control non-derepressible 2 (GCN2), which is activated during amino acid deprivation, heme-regulated inhibitor kinase (HRI), senses heme deficiency, protein kinase RNA (PKR)-like endoplasmic reticulum (ER) kinase (PERK), and gets activated during viral infections. During stress condition, these sensors get phosphorylated, which further phosphorylate the eukaryotic initiation factor (eIF2α), resulting in the attenuation of active polysome formation and global protein synthesis. The untranslated mRNA recruits different RNA-binding proteins (RBPs), which together form a structure known as “riboclusters.” These clusters dictate the fate of the mRNA transcripts (119, 120). Riboclusters formed under stress environment are also known to regulate the balance between inflammatory and anti-inflammatory cytokines through different pathways (119). ER is the primary organelle that is responsible for synthesis, folding, and trafficking of proteins. ER also participates in the detection of any metabolic changes to the cell through different cellular sensors, such as PERK, IRE1, and ATF6. Any disturbance in the normal physiology can lead to ER stress and hence activate these cellular sensors and associated stress response pathways in the form of unfolded protein response (UPR). These stress detectors are inactive under normal condition by glucose-regulated protein (GRP78), also known as BiP. Excessive stress removes the GRP78 from the sensors and makes them available for downstream response pathway. The UPR pathway responds to this stress by halting the protein translation and removing the misfolded proteins that have accumulated through chaperones. Prolonged stress can result in inflammation and related damage and ultimately, apoptosis, if the cell homeostasis is not regained (121, 122). ER stress has been implicated in various neurodegenerative and vascular diseases including different retinal degeneration diseases, Stargardt’s, age-related macular degeneration, and retinitis pigmentosa (123). Doh et al. have related ER stress with glaucoma, where high IOP stimulated the expression of BiP, CHOP, and phosphor-PERK along with ganglion cell death (124).

Prolonged stress including nutrition deprivation could also result in activation of homeostatic processes such as autophagy (125). Autophagy is a conserved defense mechanism that regulates protein turn-over as a normal phenomenon but is found to be upregulated in response to stress. Autophagy plays a crucial role in regulating immune responses including T-cell response. Coronary artery occlusion has shown to result in the increased levels of LC3-II conversion, a well-established marker for autophagy (126, 127). Different studies have investigated the role of autophagy in animal models of ischemic injury in the retina. Piras et al. demonstrated autophagy 24 h after in a retinal ischemia model induced by increasing the IOP (128). A similar outcome has been observed by Wei et al. with the accumulation of autophagosomes and high levels of LC3-II in RGCs, 6 h after the injury (129). The knowledge about cellular stress sensors and response pathways pertaining to retinal degeneration is still at an initial stage, which demands further investigations to bring out target therapies.

## Summary

Ischemia-induced retinal injury is associated with many diseases such as diabetic retinopathy and glaucoma, which result in vision impairment and blindness. With no available cure so far for retinal ischemia, it has become pertinent to identify potential targets for designing therapies. Immune modulation is one aspect that could be a potential therapeutic target for the ischemic injury. Furthermore, a better understanding about cellular stress responses can also help in future, to design better interventions for retinal ischemia–reperfusion injuries. All together, it has to be kept in consideration that since ischemia is a consequence of synergistic effects of different cell types and mechanisms; therefore, targeting a single cell or molecule will not reveal any progressive therapeutic strategy. Moreover, since ischemia is a dynamic injury, it is crucial to investigate time-dependent inflammatory responses involved so as to keep the timing for therapy in consideration with minimal damage.

## Author Contributions

GM wrote the paper; JS made the figures; and NK contributed to writing, editing, and designing of the manuscript.

## Conflict of Interest Statement

The authors declare that the research was conducted in the absence of any commercial or financial relationships that could be construed as a potential conflict of interest.

## Abbreviations

ATF6, activating transcription factor 6; BBB, blood–brain barrier; BRB, blood–retina barrier; CCL2, C–C motif chemokine ligand 2; CD, cluster of differentiation; CHOP, CCAAT-enhancer-binding protein homologous protein; CNS, central nervous system; DAMP, damage-associated molecular pattern; DEREG, depletion of regulatory T cell; eIF2α, eukaryotic initiation factor; ER, endoplasmic reticulum; GCN2, general control non-derepressible 2; GFAP, glial fibrillary acidic protein; GRP78, 78 kDa glucose-regulated protein; HMGB1, high-mobility group box 1; HRI, heme-regulated inhibitor kinase; ICAM-1, intracellular adhesion molecule 1; IFNγ, interferon gamma; IL1β, interleukin 1beta; IL18, interleukin 18; IOP, intraocular pressure; IRE1, inositol-requiring enzyme 1; ISR, integrated stress response; LC3, microtubule-associated protein light chain 3; LPS, lipopolysaccharide; MAPK, mitogen-activated protein kinases; MCAO, middle cerebral artery occlusion; mRNA, messenger ribonucleic acid; NLR, NOD-like receptor; NOD, nucleotide oligomerization domain; PAMP, pathogen-associated molecular pattern; PERK, protein kinase RNA-like endoplasmic reticulum kinase; PI3K, phosphatidylinositol-3-kinase; PKR, protein kinase RNA-activated; PRR, pattern-recognition receptor; RBPs, RNA-binding proteins; RGCs, retinal ganglion cells; ROS, reactive oxygen species; RPE, retinal pigment epithelium; SCID, severe combined immunodeficiency; TCR, T-cell receptor; TGFβ2, transforming growth factor 2; TLR, toll-like receptor; TNF-α, tumor necrosis factor-alpha; TNFR, tumor necrosis factor receptor; UPR, unfolded protein response; VEGF, vascular endothelial growth factor.
